# Impact of Sweet Potato Starch-Based Nanocomposite Films Activated With Thyme Essential Oil on the Shelf-Life of Baby Spinach Leaves

**DOI:** 10.3390/foods6060043

**Published:** 2017-06-03

**Authors:** Aseel Issa, Salam A. Ibrahim, Reza Tahergorabi

**Affiliations:** Food and Nutritional Sciences Program, Department of Family and Consumer Sciences, North Carolina Agricultural and Technical State University, Greensboro, NC 27411, USA; issaaseel@gmail.com (A.I.); ibrah001@ncat.edu (S.A.I.)

**Keywords:** antimicrobial films, sweet potato starch, *Escherichia coli*, *SalmonellaTyphimurium*, baby spinach leaves

## Abstract

*Salmonella* *Typhimurium* (*S. Typhi*) and *Escherichia coli* (*E. coli*) have been responsible for an increasing number of outbreaks linked to fresh produce, such as baby spinach leaves, in the last two decades. More recently, antimicrobial biodegradable packaging systems have been attracting much attention in the food packaging industry as eco-friendly alternatives to conventional plastic packaging. The objective of this study was to evaluate the effect of antibacterial nanocomposite films on inoculated spinach leaves and on the sensory properties of these leaves during eight days of refrigerated storage. In this study, an antibacterial film comprised of sweet potato starch (SPS), montmorillonite (MMT) nanoclays and thyme essential oil (TEO) as a natural antimicrobial agent was developed. Our results showed that the incorporation of TEO in the film significantly (*p* < 0.05) reduced the population of *E. coli* and *S. Typhi* on fresh baby spinach leaves to below detectable levels within five days, whereas the control samples without essential oil maintained approximately 4.5 Log colony forming unit (CFU)/g. The sensory scores for spinach samples wrapped in films containing TEO were higher than those of the control. This study thus suggests that TEO has the potential to be directly incorporated into a SPS film to prepare antimicrobial nanocomposite films for food packaging applications.

## 1. Introduction

The World Health Organization (WHO), the Food and Agriculture Organization of the United Nation (FAO), and the U.S. Department of Agriculture (USDA) all recommend produce consumption for consumers due to the presence of high levels of micronutrients and fibers that can reduce the risk of cardiovascular diseases and cancer [1]. However, the food safety of fresh produce is a matter of increasing concern because these foods usually receive minimal processing and are often consumed as a ready-to-eat product. In the U.S., there were more cases of foodborne illnesses associated with fresh produce than those involving seafood, poultry, beef, pork, or eggs [2]. *Salmonella* spp., *Escherichia coli* O157:H7, *Listeria monocytogenes*, and *Shigella* spp. have been frequently associated with illness outbreaks related to the consumption of fresh produce, respectively [2]. An analysis of outbreak data in the U.S. from 1988 to 2008 shows that there was an average of 6.3–13.2 illness outbreaks each year caused by produce. More recently, leafy greens have been responsible for one third of these same types of outbreaks [3].

Fresh-cut baby spinach leaves have a very high respiration rate and require high levels of O_2_ in their packaging in order to maintain their quality [4]. Commercially processed baby spinach leaves are currently packaged with perforated films to allow adequate O_2_. This practice, however, raises a food-safety concern that human pathogenic bacteria could transfer from the environment through the perforations and result in post-processing contamination of the produce [4]. Thereby, it is essential that a packaging system be developed to improve the safety, as well as to maintain the sensory properties, of fresh-cut baby spinach leaves.

The primary types of plastics that are currently used in all applications are derived from non-renewable petroleum resources and are non-biodegradable. The volume of plastics discarded annually creates a substantial waste disposal issue. In a 2005 report, 28.9 million tons of plastic packaging was generated in the U.S., and only approximately 5.7% of plastic was recycled or reused in some way. The remaining 94.3% was sent to landfills and discarded or combusted into the environment [5]. Therefore, alternative strategies such as the sustainable use of natural and renewable resources to reduce the quantity of persistent plastic waste are warranted.

Recently, biodegradable films made of biopolymers have become more attractive for food packaging as an alternative to typical commercial plastic packaging [6]. Many types of biopolymers such as corn starch, wheat gluten, chitosan, soy, and whey proteins have been used for biodegradable packaging. However, there is potential allergenicity if these compounds migrate onto edible products [7,8,9]. Sweet potato starch can be used to overcome this issue as there has been no report of allergenicity associated with sweet potatoes thus far. Sweet potato (*Ipomoea batatas* Lam) is an inexpensive and readily available vegetable that is cultivated extensively for its nutritious value across many regions of the world. Sweet potato starch (SPS) with a 58–76% starch content (on a dry basis), has properties that are similar or better than those of potato starch [10]. However, sweet potato does not have the mechanical and barrier properties matching those of plastics [11]. The addition of nano-scale particles into starch can change crystallization kinetics, crystalline morphology, crystal forms, and crystallite size. As a result, the addition of these particles may improve the mechanical and barrier properties of starch. By far the most promising nanoscale fillers for biodegradable packaging are montmorillonite (MMT) nanoclays. The popularity of MMT nanoclays in food contact applications derives from their low cost, effectiveness, and high stability [12]. The incorporation of MMT into starch matrices has been used to enhance mechanical and barrier properties [13].

Nanocomposite films are also excellent vehicles for incorporating a wide variety of additives such as antioxidants and antimicrobial agents. The effect of these additives may be an improvement in food quality and safety. The antibacterial activities of films are determined in part by the release rate of antibacterial agents. If the release is too slow and the microbial growth is not sufficiently inhibited, the release is too fast and the inhibition is not sustained [14]. MMT nanoclay is a compound that can potentially be used to control the release of antimicrobial agents from film materials.

Natural antimicrobial agents have attracted increasing attention as a replacement for synthetic ones in the food packaging industry. Several natural antimicrobials have been incorporated into food packaging such as plant essential oils (EO), nisin and chitosan (a natural biopolymer extracted from crustacean shells). However, nisin has a limited spectrum of activity, does not inhibit Gram-negative bacteria or fungi, and is only effective at a low pH [15]. In addition, there are concerns with the allergenicity of chitosan incorporated into food packaging [16]. Among EOs, thyme (*Thymus vulgaris*) EO (TEO) has received the most attention from researchers. Thymol, the major component of TEO, has been found to possess antimicrobial activity in vitro against a broad spectrum of bacteria, such as *S*. *Typhimurium*, *L. monocytogenes* [17] *Escherichia coli*, *Pseudomonas fluorescens*, *Staphylococcus aureus*, *Lactobacillus plantarum*, and *Bacillus subtilis*, as well as *Shigella sonnei* and *Shigella flexneri* [18]. Thymol is considered to present no risk to the health of consumers, has been registered by the European Commission [19], and is generally recognized as safe (GRAS) by the Food and Drug Administration (FDA) [3].

Studies related to SPS as a source of starch for film packaging are very limited. In addition, up to now, there is no published report related to the application of an antibacterial SPS-based bio-nanocomposite film to a real food system or fresh produce. Thus, the overall objective of this study was to develop biodegradable nanocomposite-based food packaging films which incorporate TEO and MMT nanofillers with SPS to address issues of food safety, the environmental impact, and agricultural sustainability. The specific objectives of the research were to: (1) Develop biodegradable MMT nanocomposite films with SPS by incorporating TEO, (2) Investigate the antibacterial activity of films on inoculated baby spinach leaves, and (3) Evaluate the sensory properties of baby spinach leaves during refrigerated storage.

## 2. Materials and Methods

### 2.1. Bacterial Strains

Stock cultures of *strains* of *E. coli K-12* (ATCC 25253) and *S. Typhimurium* (ATCC 53648) were obtained from the American Type Culture Collection. The microorganisms were kept frozen at −80 °C in tryptic soy broth (TSB) containing glycerol (10%, *v/v*). Before use, the stock cultures were resuscitated through two consecutive 24-h growth cycles in TSB at 37 °C to obtain working cultures containing approximately 10^5^ colony forming unit (CFU)/mL, as determined by serial dilution and a plate count.

### 2.2. Antibacterial Activity of Thyme Essential Oil

The antibacterial properties of TEO were studied using the agar diffusion method as reported by [20]. Initially, the plates were seeded with 0.1 mL of inoculum by swab containing approximately 10^5^ CFU/mL of the indicated bacteria. Then, an agar well (with 5 mm diameter) was created with a sterile core bore on the agar. Lastly, thirty µL of TEO was poured into the well. The plates were incubated at 37 °C for 24 h in an incubation chamber. The diameter of the resulting zone of inhibition was measured in mm.

### 2.3. Preparation of SPS/Clay-TEO Solution

The SPS/clay-TEO solution was prepared according to the previously described method with a slight modification [11]. An aqueous solution of SPS was prepared by dissolving 50 g of SPS in 1000 mL dH_2_O, moderately stirred at room temperature, and then heated to 80 °C for 30 min. After gelatinization, glycerol (Glycerol, Fisher Scientific, G33-1, Fair Lawn, New Jersey) was added as a plasticizer at a concentration of 30% (*w/w*, on dry basis of the weight of starch) and the resulting dispersion was subjected to further mixing for 5 min. MMT (Montmorillinite, Nanomer, St. Louis, MO, USA) powder (3% *w/w* of SPS) was separately dispersed into 60 mL of distilled water under magnetic stirring at 500 rpm for 48 h. Then, the resulting dispersion was added to the SPS-glycerol suspension solution and the mixture underwent high shear lab mixing at 5000 rpm for 10 min. Finally, the thyme essential oil (*Thymus Vulgaris*, New Direction Aromatics, Mississauga, ON, Canada), previously mixed with Tween 80 (Fisher Scientific, Fair Lawn, NJ, USA) (0.25 g/g of essential oil) to help create a uniform and stable distribution, was incorporated into the film forming solution at several concentrations (0, 2, 4, and 6% *v/v* on the basis of neat film solution). Samples were then homogenized at 20,000 rpm for 5 min using a laboratory homogenizer (Homogenizer, OMNI International, Kenneswa, GA, USA), after being degassed using an ultrasonic bath (Branson sonifier, Model 3800, Danbury, CT, USA).

### 2.4. Preparation of Nanocomposite Films

The films were developed by means of a casting process in which the dispersion solution (60 mL) was spread over a Teflon plate and then dried for 24 h at 23 ± 2 °C and 50 ± 5% relative humidity (RH). The dried films were peeled off the plates and stored in a desiccator containing a saturated magnesium nitrate solution at 25 °C and 52% RH until use. All films consisted of the same amounts of distilled water, SPS, MMT, and glycerol, but different proportions of TEO. One treatment without TEO and one treatment without TEO and MMT were considered as the control.

### 2.5. Antibacterial Activity of the Film

The antimicrobial test was carried out according to the method developed by [21]. An agar diffusion assay was used to determine the antibacterial activity of the SPS nanocomposite film. Mueller-Hinton agar (MHA) medium (Remel, Lenexa, KS, USA) was used to ensure better antimicrobial disk diffusion. Each culture of *E. coli* and *S. Typhimurium* was streaked onto an MHA medium. Circular discs (5 mm diameter) were cut from the nanocomposite films containing 0, 2, 4, and 6% (*v/v*) TEO. One circular disc obtained from each film was placed on top of the MHA medium inoculated with pathogenic bacteria, and the plates were incubated at 37 °C for 24 h. The film without TEO was used as a control. After incubation, the plates were examined to find the zone of inhibition of the film discs. The diameters of the inhibition zones were measured by subtracting the diameter of the entire inhibition zone (mm) from the diameter of the disc.

### 2.6. Antimicrobial Effects of Films on Inoculated Baby Spinach Leaves During Refrigerated Storage

#### 2.6.1. Baby Spinach Leaves Preparation

Baby spinach leaves were purchased from a local grocery store one day before testing. Spinach leaves were surface sterilized by immersion in 70% methanol for 30 s, rinsed in sterile distilled water, and allowed to air-dry. Sterilized leaves were inoculated by pipetting 100 µL of ~10^5^ CFU/mL bacterial suspension on to the surface of each leaf. The inoculated leaves were then held under a laminar flow biological hood at room temperature for 1 h to allow inoculum drying and attachment before further treatment [22]. After drying, the baby spinach leaves were wrapped with the film and stored at 4 °C for eight days. The populations of *E. coli K-12* and *S. Typhimurium* bacteria in the samples were determined immediately following inoculation and periodically at two-day intervals during the storage period. All tests were done in triplicate.

#### 2.6.2. Microbiological Analysis of Spinach Samples

Two controls were used: (1) An uninoculated, un-sterilized, and unwrapped control, to determine the background microflora **(**total viable count, yeast, and molds); and (2) An inoculated and unwrapped control for comparison with inoculated and wrapped spinach samples. Baby spinach samples (25 g each) in bio-nanocomposite films were aseptically placed in polyethylene stomacher bags (PE bags, Fisher Scientific Co., Fair Lawn, NJ, USA) and macerated with 225 mL of 0.1% (*w/v*) peptone water for 2 min. The macerated samples were filtered through sterile glass wool, serially diluted in peptone water, and surface plated onto the MHA medium. Plates were incubated at 35 °C for 24 to 48 h, and colonies of *E. coli K-12* and *S. Typhimurium* were counted.

### 2.7. Sensory Evaluation of Baby Spinach Leaves

A sensory panel employed to test the acceptability of the uninoculated baby spinach leaves wrapped in nanocomposite films was conducted by a group of untrained panelists. The following sensory parameters were investigated: (a) odor, (b) color, and (c) overall acceptability. The descriptions for each score were as follows; 9 = like extremely or extremely high, 8 = like very much or very high, 7 = like moderately or high, 6 = like slightly or lightly high, 5 = neither like or dislike or neither high or low, 4 = dislike slightly or slightly low, 3 = dislike moderately or low, 2 = dislike very much or very low, and 1 = dislike extremely or extremely low. Testing was carried out in a sensory analysis laboratory with appropriate lighting conditions and a temperature of around 20 °C. The study was approved by the Institutional Review boards (IRB) at NC A&T State University.

### 2.8. Statistical Analysis

The data were presented as the mean ± standard deviation of each treatment. All samples were run in triplicate. The experiments were factorial with a completely randomized design using a two- way analysis of variance (ANOVA) using the SAS program (version 8.1; 2002, Statistical Analysis System Institute Inc., Cary, NC, USA). Differences between the mean values were compared using Tukey’s range test and a probability value of *p* < 0.05 was considered significant.

## 3. Results

### 3.1. Antimicrobial Activity of the Pure Thyme Essential Oil

**Figure 1:**
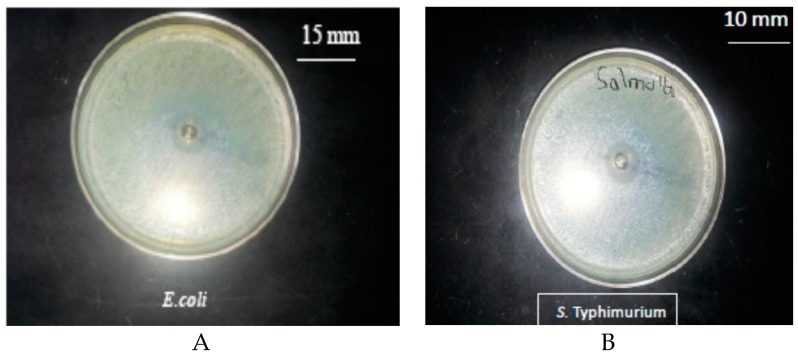
Antibacterial activity of pure thyme essential oil (TEO) against (**A**) *Escherichia coli*, (**B**) *Salmonella Typhimurium.*

The antimicrobial activity of pure thyme (*Thymus vulgaris*) essential oil (TEO) against the selected microorganisms, using the agar well diffusion test, is presented in Figure 1. The results indicated that both *E. coli* and *S*. *Typhimurium* were sensitive to TEO. TEO inhibited the growth of bacterial strains by producing inhibition zone diameters of 15 mm and 10 mm for *E. coli* and *S*. *Typhimurium* respectively.

### 3.2. Antimicrobial Activity of Films

**Figure 2:**
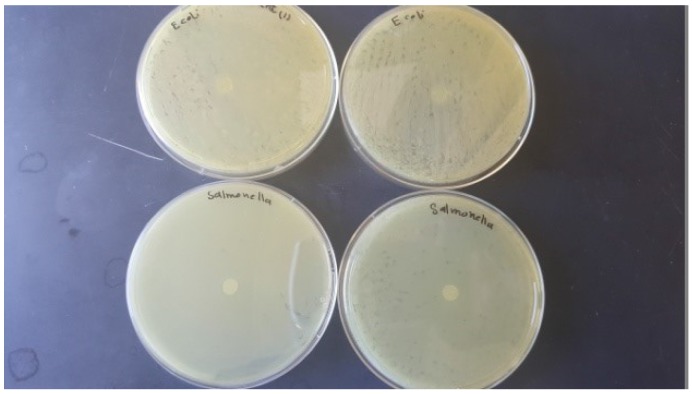
Antibacterial activity of the neat sweet potato starch-based films against *Escherichia coli* and *Salmonella Typhimurium*.

**Figure 3:**
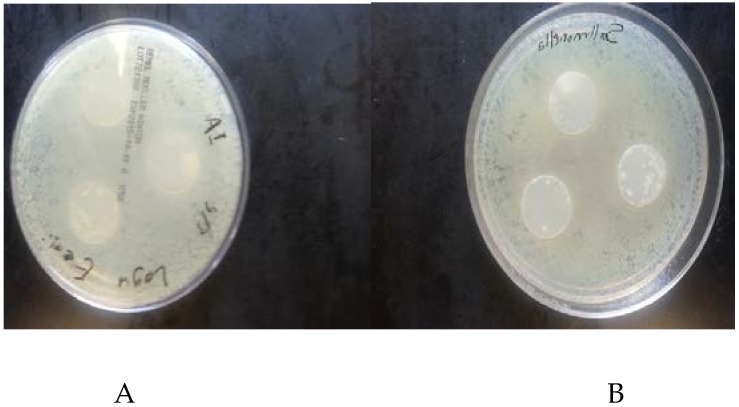
Antibacterial activity of sweet potato starch-based nanocomposite films with montmorillonite nanoclay and without thyme essential oil, agaisnt (**A**) *Escherichia coli* (**B**) *Salmonella Typhimurium*.

**Figure 4:**
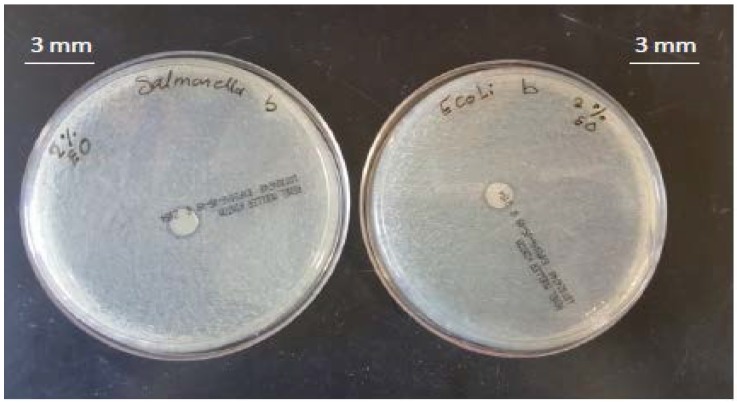
Antibacterial activity of sweet potato starch-based nanocomposite films with montmorillonite nanoclay and 2% (*v/v*) thyme essential oil against *Escherichia coli* and *Salmonella Typhimurium*.

**Figure 5:**
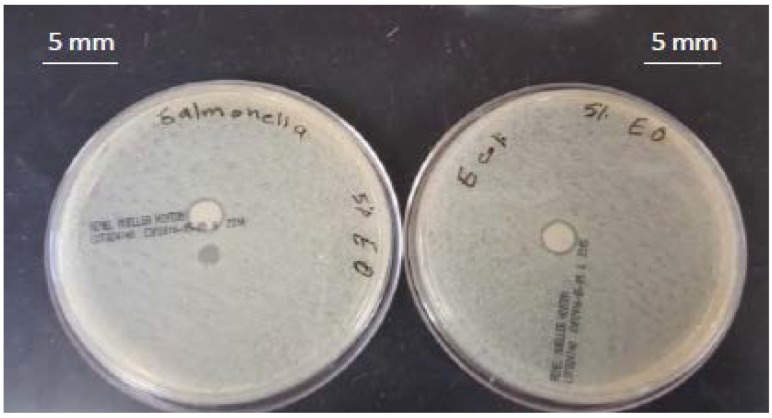
Antibacterial activity of sweet potato starch-based nanocomposite films with montmorillonite nanoclay and 4% (*v/v*) thyme essential oil against *Escherichia coli* and *Salmonella Typhimurium*.

**Figure 6:**
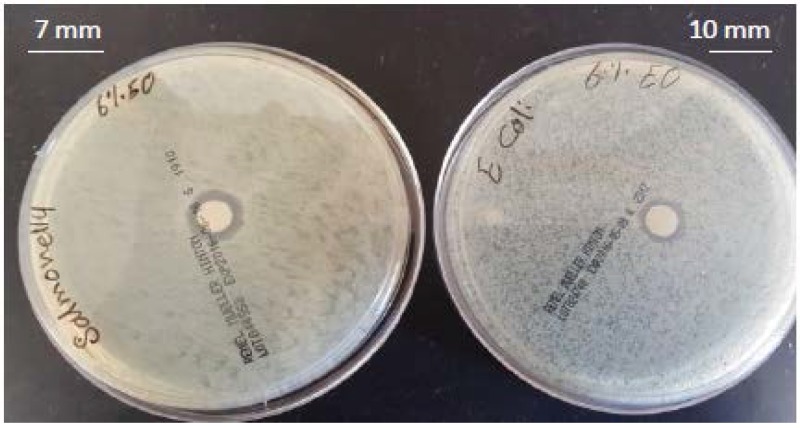
Antibacterial activity of sweet potato starch-based nanocomposite films with montmorillonite nanoclay and 6% (*v/v*) thyme essential oil against *Escherichia coli* and *Salmonella Typhimurium*.

The antimicrobial activities of SPS-based films are shown in Table 1 and Figure 2, Figure 3, Figure 4, Figure 5 and Figure 6. When antimicrobial agents are incorporated into food packaging films, these materials diffuse through the agar gel and this results in a clear zone around the film cuts. Our result indicated that a neat SPS film has no antibacterial properties. Additionally, MMT nanoclay did not exhibit any antibacterial activities in the agar diffusion test.

The incorporation of TEO in the bio-nanocomposite films at levels higher than 2% (*v/v*) exhibited a clear inhibitory zone, as evidenced by the absence of bacterial growth around the film cuts. The highest concentration of TEO (6%) resulted in the maximum bacterial growth inhibition.

### 3.3. The Effect of Films on Total Viable Count, Yeast and Mold Growth of Baby Spinach Leaves

Table 2 shows the changes in the total viable count (TVC), and yeast and mold counts of baby spinach leaves either unwrapped or wrapped with films containing 0, 2.0, 4.0, and 6.0% TEO over eight days of refrigerated storage. The initial number of TVC in baby spinach samples was 2.80 log CFU/g, which is an indication of the high quality of spinach used in this study. According to the literature, the bacterial counts of bagged cut spinach are around 7.0 log CFU/g, with a broad range of <4 to 8.3 log CFU/g [23]. Table 2 shows that the TVC and yeast/mold of all un-wrapped samples increased with storage time. The baby spinach samples wrapped in neat SPS films without TEO showed a decrease in bacterial, yeast, and mold populations. Films containing 2% TEO showed a lower number of microorganisms compared to the control samples after storage at 4 °C for two and eight days. However, when baby spinach samples were wrapped in films with 4 and 6% TEO, no bacterial or yeast and mold growth was detected during the storage period.

### 3.4. The Antibacterial Effect of the Films on Baby Spinach Leaves Inoculated with E. coli and S. Typhimurium

The microbiological changes in baby spinach leaves stored at 4 °C are shown in Table 3. The initial population of *E. coli* and *Salmonella* in unwrapped baby spinach leaves increased substantially from the initial inoculation level of 2.9 to 4.8 and 2.3 to 6.8 log CFU/g, respectively, by the end of the experiment. The bacterial population of samples wrapped in the SPS-based nanocomposite with TEO either decreased or was not detectable by the end of the storage period. TEO was shown to be very effective at controlling the growth of *Salmonella* and *E. coli* in the highest concentration of TEO (6%), and a total inhibition of pathogen growth occurred during the storage period. In the presence of 4% TEO, a complete inhibition of microbial growth was observed during the entire time of storage, and this film reduced the population of *E. coli* and *Salmonella* by more than 4.5 log CFU/g compared to the control. Consequently, the nanocomposite containing TEO increased the shelf-life of the spinach leaves by about eight days as compared to the control.

### 3.5. Sensory Evaluation of Baby Spinach Leaves

A sensory evaluation (color, odor, and overall appearance) of the samples of wrapped spinach leaves was investigated in this study. The results for the sensory evaluation of baby spinach leaves wrapped in nanocomposite films at different days during the storage period are presented in Table 4. There was no significant difference in the overall appearance between the wrapped and unwrapped samples (*p* > 0.05). However, the overall appearance of wrapped samples with nanocomposite films incorporated with different concentrations of TEO was rated slightly higher than that of unwrapped samples. Samples wrapped with the highest TEO concentration (6%) had numerically higher appearance values compared to the control samples. With regard to odor and color, no significant differences (*p* > 0.05) were observed between wrapped samples and control samples. In general, wrapped samples had a better odor, color, and appearance than unwrapped samples, which could probably be attributed to the aromatic effect of thyme essential oil.

## 4. Discussion

### 4.1. Antimicrobial Activity of Pure Thyme Essential Oil

Various studies have shown variations in the antimicrobial properties of plant essential oils. These variations might be explained by the differences in the susceptibility of testing conditions, the culture media chosen for microbial growth, and the selected microorganisms chosen for the evaluation [24]. Additionally, there is a relationship between the chemical composition of the most abundant compounds in essential oil and the resultant antimicrobial activity. The major constituents of the *T. vulgaris* oil used in this study were thymol (57.7%), p-cymene (18.7%), and carvacrol (2.8%). Researchers [25] tested different species of thyme essential oil and determined that a greater efficacy was achieved when essential oils from *T. hyemalis*, followed by *T. zygis* and *T. vulgaris*, were used. This could be due to the fact that most of the antimicrobial activity in essential oils from the genus Thymus appears to be associated with phenolic compounds (thymol and carvacrol), since the contents of thymol and carvacrol in *T. zygis* and *T. hyemalis* were higher than those of *T. vulgaris*. This result agrees with those reported by other authors [26,27,28].

### 4.2. Antimicrobial Activity of the Films

The neat SPS film did not exhibit any antibacterial properties. These results correlate with the results of one similar study [11]. This effect may be related to the fact that SPS does not diffuse through the adjacent agar media in the agar diffusion test method; as a result, only microorganisms in direct contact with the active sites of SPS are inhibited. Additionally, MMT did not show antibacterial activity in the agar diffusion test and this result is in agreement with that of researchers [29] who tested MMT nanoclay in the well. They reported that MMT did not show any antibacterial activities.

Others [21] reported that the use of 2% thyme essential oil in a whey protein isolate film was the minimum inhibitory level against *Staphylococcus aureus*, *Salmonella enteritidis*, *Listeria monocytogenes*, *Lactobacillus plantarum*, and *Escherichia coli* O157:H7. This correlates with the results in our study. As previously discussed, the antimicrobial activity of thyme has been attributed to its essential oils, which contain the terpenes: carvacrol and thymol. According to a review [19], carvacrol and thymol break down the outer membrane of microorganisms, which leads to an excessive leakage of essential elements causing bacterial death. In general, in gram-negative bacteria, the outer membrane constitutes the outer surface of the cell wall, leading to a decrease in the effectiveness of antimicrobial agents. In addition, the effect of TEO refers to its ability to increase the permeability of the outer membrane of the cell, leading to a release of lipopolysaccharides and an increase in ATP loss [30].

In addition to the film preparation method, the source and concentration of active components of plant essential oils and basic film material have a crucial effect on the antimicrobial activity of films. The release of antimicrobial agents from the films is also dependent on many factors, including electrostatic interactions between the antimicrobial agent and the polymer chains, osmosis, structural changes induced by the presence of the antimicrobial, and environmental conditions [31].

In general, films containing TEO are very hydrophilic; thus, they absorb water quickly, which results in swelling. As a result, the active components in these films migrate very fast. As the concentration of essential oil increases, the zone of inhibition also increases. The largest inhibition zones were observed when 6% (*v/v*) of TEO was incorporated into the film. Of the bacteria examined, *Salmonella* showed a higher resistance, while *E. coli* was more sensitive to TEO containing films with an inhibition zone of 10 mm. These results correlate with the results of other studies [32,33], which indicated that the antibacterial effect of essential oils depended on the concentration of essential oil.

### 4.3. The Effect of Films on Total Viable Count, Yeast and Mold Growth of Baby Spinach Leaves

The baby spinach samples wrapped in neat SPS films without TEO showed a decrease in bacterial, yeast, and mold populations. This might be due to the superior oxygen barrier properties of starch-based films, which limit the growth of aerobic bacteria on samples wrapped in films [11]. Films containing 2% TEO showed a lower number of microorganisms compared to the control samples after storage at 4 °C for two and eight days. However, when the baby spinach samples were wrapped in films with 4 and 6% TEO, no bacterial or yeast and mold growth was detected over the storage period. This indicates that TEO incorporated in SPS nanocomposite films can strongly inhibit the growth of the microorganisms. Researchers [34] reported a reduction in the initial total viable counts of Asian sea bass samples with the addition of 0.05% of oregano and/or thyme EO.

### 4.4. The Antibacterial Effect of Films on Baby Spinach Leaves Inoculated with E. coli and S. Typhimurim

The bacterial population of samples wrapped in the SPS-based nanocomposite with TEO either decreased or was undetectable by the end of the storage period. Similar results were obtained by others [29], who studied the effect of a bionanocomposite from chitosan and rosemary essential oil on sliver carp fillets. As previously discussed, the antimicrobial activity of thyme has been attributed to its EOs, which contain terpenes: carvacrol (2-methyl-5-(1-methylethyl) phenol) and thymol (5-methyl-2-(1-methylethyl) phenol), respectively. The antimicrobial activity of cinnamon, oregano, and thyme EOs against various Gram-negative bacteria (*E. coli*, *Y. enterocolitica*, *P. aeruginosa,* and *Sal. choleraesuis*), Gram-positive bacteria (*L. monocytogenes*, *Staph. aureus*, *B. cereus*, and *E. faecalis*), yeasts (*C. albicans*), and molds (*A. flavus*, *P. islandicum*) has been reported [33]. TEO was shown to be particularly effective against all bacteria during incubation. By comparison, the results of our study indicated that when TEO was incorporated into the film matrix, the antimicrobial activity of TEO was maintained, but exhibited less antibacterial activity in films in comparison with pure essential oil. This result might be the due to the lower amount of EO in the film solution in comparison with the well test for pure EO, as well as the partial loss of volatile compounds during film preparation [34]. Essential oils contain around 85–99% volatile and 1–15% non-volatile components [35].

It is notable that, as the storage time increased, the film effectiveness increased. As shown in Table 3, in all pathogens, growth was reduced by the end of the storage period. As previously discussed, the antibacterial activities of films are determined in part by the release rate of antibacterial agents. If the release is too slow, the microbial growth is not inhibited sufficiently; conversely, if the release is too fast, inhibition will not be sustained [14]. This effect could be due to the contribution of MMT nanoclay, which has the potential to control the release of antimicrobial agents in film materials.

### 4.5. Sensory Evaluation of Baby Spinach Leaves 

A sensory evaluation consists of a set of techniques for the accurate measurement of human reaction to the odor, texture, and flavor of foods [36]. A sensory evaluation can provide useful information to product developers and food scientists about their products’ sensory appeal to consumers [37]. In general, wrapped samples had a better odor, color, and appearance than unwrapped samples, which was probably due to the aromatic effect of thyme essential oil.

## 5. Conclusions

A combination of sweet potato starch, MMT nanoclay, and thyme essential oil (TEO) was used to develop an active biodegradable nanocomposite film for food packaging. The extent to which this film enhanced the shelf life of baby spinach leaves stored at a refrigerated temperature during eight days was studied. The results confirmed that films containing TEO could effectively improve the acceptability of samples by around eight days compared with the control. Moreover, the film had no adverse effects on the sensory properties of the product. Consumer preferences were greater for films with higher TEO concentrations due to the more desirable odor, color, and overall acceptability of the spinach. Therefore, this study demonstrated the effectiveness of SPS/clay films containing TEO in controlling the growth of *Escherichia coli* and *Salmonella Typhimurium* and improving the microbiologcal quality of baby spinach leaves during refrigerated storage. Further studies should be directed towards underestanding how SPS-based films could impact the quality, safety and shelf-life of different fresh produce. In the near future, our group will report the results of the phyisco-mechancial aspects of SPS-based nanocomposites.

## Figures and Tables

**Table 1:** Antibacterial activity of sweet potato starch-based (SPS-MMT) nanocomposites activated by thyme essential oil (TEO) at different levels against *Escherichia coli* and *Salmonella Typhimurium.*

Treatment	MMT (%)	TEO (%)	Inhibition Zone (mm)	
			*E. coli*	*S. Typhymurium*
1	0	0	0	0
2	3	0	0	0
3	3	2	3	3
4	3	4	5	5
5	3	6	10	7

**Table 2:** Antimicrobial effect of sweet potato starch-based nanocomposites activated by thyme essential oil at different levels on the total viable count (TVC), and yeast and molds during eight days of refrigerated storage.

Day	Microorganisms	Unwrapped	Treatment				
			1	2	3	4	5
			Log Colony Forming Unit (CFU)/g				
Day 2	TVC	2.80	2.50	2.34	1.00	1.00	0.69
	Yeast/Mold	1.70	1.63	0.5	ND	ND	ND
Day 8	TVC	3.00	2.04	1.00	ND	ND	ND
	Yeast/Mold	1.80	1.75	ND	ND	ND	ND

TVC—Total viable count, ND—Non detectable.

**Table 3:** *Antibacterial* activity of sweet potato starch-based nanocomposites activated by thyme essential oil at different levels on innoculated baby spinach leaves with *Escherichia coli* and *Salmonella Typhimurium* during eight days of refrigerated storage.

Treatment	Microorganism	Log CFU/g				
			Day 2	Day 4	Day 6	Day 8
1	*E. coli*	Unwrapped	1.60	1.60	2.47	3.00
		wrapped	1.20	1.20	1.15	1.10
	*Salmonella*	Unwrapped	2.04	2.95	2.25	6.80
		wrapped	1.08	ND	ND	ND
2	*E. coli*	Unwrapped	2.00	2.90	3.60	3.75
		wrapped	1.30	ND	ND	ND
	*Salmonella*	Unwrapped	1.69	2.60	2.80	3.00
		wrapped	1.60	1.00	ND	ND
3	*E. coli*	Unwrapped	2.07	1.00	4.00	4.30
		wrapped	1.30	ND	DN	DN
	*Salmonella*	Unwrapped	1.30	1.00	3.00	3.69
		wrapped	DN	DN	DN	DN
4	*E. coli*	Unwrapped	2.84	2.90	3.00	3.00
		wrapped	DN	DN	DN	DN
	*Salmonella*	Unwrapped	2.84	3.23	3.96	4.30
		wrapped	DN	DN	DN	DN
5	*E. coli*	Unwrapped	2.90	2.95	3.07	4.84
		wrapped	DN	ND	DN	DN
	*Salmonella*	Unwrapped	2.30	2.47	4.00	4.90
		wrapped	ND	ND	DN	DN

ND—Non detectable.

**Table 4:** Sensory properties of baby spinach leaves wrapped in sweet potato starch-based nanocomposites activated by thyme essential oil at different levels during eight days of refrigerated storage.

Days	Unwrapped	Treatment				
		1	2	3	4	5
		color				
1	8.0 ± 0.00 ^a^	8.0 ± 0.00 ^a^	8.0 ± 0.00 ^a^	9.0 ± 1.00 ^a^	8.0 ± 0.00 ^a^	8.5± 0.70 ^a^
8	7.0 ± 0.01 ^a^	7.0 ± 0.03 ^a^	7.0 ± 0.82 ^a^	7.0 ± 0.96 ^a^	7.0 ± 0.5 ^a^	7.5 ± 0.58 ^a^
		Odor				
1	7.0 ± 0.02 ^a^	7.5 ± 0.00 ^a^	7.5 ± 0.00 ^a^	8.0 ± 0.50 ^a^	8.0 ± 0.70 ^a^	8.0 ± 0.70 ^a^
8	7.0 ± 0.01 ^a^	7.0 ± 0.00 ^a^	7.0 ± 0.00 ^a^	7.0 ± 0.00 ^a^	7.5 ± 0.70 ^a^	7.5 ± 0.70 ^a^
		Overall appearance				
1	8.0 ± 0.00 ^a^	8.0 ± 0.01 ^a^	8.0 ± 0.00 ^a^	8.0 ± 0.01 ^a^	8.5 ± 0.05 ^a^	8.0 ± 0.04 ^a^
8	8.0 ± 0.00 ^a^	7.0 ± 0.00 ^a^	7.0 ± 0.00 ^a^	8.0 ± 0.00 ^a^	8.0 ± 0.70 ^a^	8.5 ± 0.70 ^a^

Data are given as mean values ± standard deviation (*n* = 3). Different letters within the same row indicate significant differences (Tukey’s Test, *p* < 0.05) between mean values.
